# Reactivation of Endogenous Genes and Epigenetic Remodeling Are Barriers for Generating Transgene-Free Induced Pluripotent Stem Cells in Pig

**DOI:** 10.1371/journal.pone.0158046

**Published:** 2016-06-23

**Authors:** Kwang-Hwan Choi, Jin-Kyu Park, Dongchan Son, Jae Yeon Hwang, Dong-Kyung Lee, Hakhyun Ka, Joonghoon Park, Chang-Kyu Lee

**Affiliations:** 1 Department of Agricultural Biotechnology, Animal Biotechnology Major, and Research Institute of Agriculture and Life Science, Seoul National University, Seoul, Korea; 2 Division of Animal Sciences and Bond Life Sciences Center, University of Missouri, Columbia, Missouri, United States of America; 3 Department of Biological Resources and Technology, Yonsei University, Wonju, Korea; 4 LG Life Sciences, R&D Park, Daejeon, Republic of Korea; 5 Institute of Green Bio Science and Technology, Seoul National University, Pyeong Chang, Kangwon do, Korea; University of Tampere, FINLAND

## Abstract

Cellular reprogramming of committed cells into a pluripotent state can be induced by ectopic expression of genes such as OCT4, SOX2, KLF4, and MYC. Reprogrammed cells can be maintained by activating endogenous pluripotent networks without transgene expression. Although various research groups have attempted to generate pig induced pluripotent stem cells (iPSCs), authentic iPSCs have not be obtained, instead showing dependence on transgene expression. In this study, iPSCs were derived from porcine fetal fibroblasts via drug-inducible vectors carrying human transcription factors (OCT4, SOX2, KLF4, and MYC). Therefore, this study investigated characteristics of iPSCs and reprogramming mechanisms in pig. The iPSCs were stably maintained over an extended period with potential *in vitro* differentiation into three germ layers. In addition, the pluripotent state of iPSCs was regulated by modulating culture conditions. They showed naive- or primed-like pluripotent states in LIF or bFGF supplemented culture conditions, respectively. However, iPSCs could not be maintained without ectopic expression of transgenes. The cultured iPSCs expressed endogenous transcription factors such as OCT4 and SOX2, but not NANOG (a known gateway to complete reprogramming). Endogenous genes related to mesenchymal-to-epithelial transition (*DPPA2*, *CDH1*, *EPCAM*, and *OCLN*) were not sufficiently reactivated, as measured by qPCR. DNA methylation analysis for promoters of OCT4, NANOG, and XIST showed that epigenetic reprogramming did not occur in female iPSCs. Based on our results, expression of exogenous genes could not sufficiently activate the essential endogenous genes and remodel the epigenetic milieu to achieve faithful pluripotency in pig. Accordingly, investigating iPSCs could help us improve and develop reprogramming methods by understanding reprogramming mechanisms in pig.

## Introduction

Pluripotent stem cells (PSCs) are a promising tool for human regenerative medicine, and in domestic animals, as useful tools for producing transgenic and disease model animals. PSCs have two distinct grades of pluripotency, “naïve” and “primed” states, based on developmental competence [1]. Naïve PSCs, represented by mouse embryonic stem cells (ESCs) and embryonic germ cells (EGCs), are characterized by dome-shaped colony morphologies, activation of LIF signaling, and two active X chromosomes in females. By contrast, primed PSCs, including epiblast stem cells (EpiSCs), are defined by flattened colony morphologies and activated FGF signaling pathways. Compared with the primed state, naïve PSCs have developmental and functional ground states showing contributions to blastocyst chimeras and higher transgenic efficiency [2, 3]. For these reasons, many research groups have attempted to generate naïve PSCs of domestic animals (especially pig) for the efficient production of transgenic animals [4–7].

PSCs have been derived from early epiblasts in preimplantation blastocysts, which are known as ESCs. Induced pluripotent stem cells (iPSCs), as an alternative source of ESCs, can be generated by Yamanaka’s factors [8]. The acquisition of pluripotency in fibroblasts is accomplished by genetic and epigenetic events termed initiation, maturation, and stabilization [9]. The initiation of reprogramming is defined by mesenchymal-to-epithelial transition (MET), in which epithelial-specific genes are upregulated and TGFB1 is downregulated by expression of reprogramming factors and BMP signaling [9, 10]. Subsequently, NANOG and SALL4 induced by SOX2 activate endogenous pluripotent networks, and pluripotent circuitry is stabilized via epigenetic remodeling such as DNA methylation, histone modification, and X chromosome reactivation [11, 12]. Finally, the reprogrammed cells can be maintained without ectopic expression of transgenes, which indicates that endogenous pluripotent network are fully activated and stabilized [13, 14].

As described above, several genetic and epigenetic changes occur during nuclear reprogramming from somatic cells to iPSCs. Achieving faithful pluripotency is required to overcome epigenetic and physiological obstacles such as the epigenetic memory of somatic cells [15, 16], MET [10], repressive chromatin [17], and apoptosis and cell cycle arrest [18, 19]. However, if these barriers are not overcome, silencing of transgenes, epigenetic remodeling, and lack of NANOG expression occur, resulting in partial reprogramming of iPSCs (pre-iPSCs) [20, 21]. Although various research groups have attempted to generate pig iPSCs (piPSCs), authentic iPSCs have not be obtained, instead showing features of incomplete reprogramming including dependence on transgene expression, epigenetic remodeling, reactivation of pluripotent genes, and chimera formation [22–27]. This may be due to differences in the molecular mechanisms during embryo development between mouse and pig [28]. It is important to investigate pre-iPSCs to improve and develop reprogramming methods by understanding reprogramming mechanisms in pig.

Pig has been identified as a valuable candidate model animal for human disease models and xenotransplantation, because of physiological and anatomical similarity between human and pig. In addition, the establishment of authentic ESCs and iPSCs, which can maintenance pluripotency without ectopic expression and have *in vivo* differentiation ability, is important in pigs for medical and industrial usages. Here, we derived several piPSC lines by introducing Yamanaka’s factors using drug-inducible vectors. These cell lines were incompletely reprogrammed, not meeting the criteria of PSCs such as pluripotent gene expression. Accordingly, we explored the state where pig iPSCs committed to pluripotency through genetic and epigenetic analyses. We verified that failures of MET and epigenetic remodeling were occurred in pig pre-iPSCs during reprogramming. Expression of exogenous genes could not sufficiently activate the essential endogenous genes for reprogramming into pluripotency in pig. Consequently, further in-depth analyses of pig-specific signaling pathways are required to establish authentic porcine embryonic stem cells and obtain completely reprogrammed and transgene-free iPSCs.

## Materials and Methods

### Animal welfare

The care and experimental use of pigs and mice was approved by the Institute of Laboratory Animal Resources, Seoul National University (SNU-140501-4, SNU-140422-3 and SNU-140328-2). A pregnant sow was purchased from animal farm. The sow was taken care exclusively at farm and sacrificed after 27 days from artificial insemination at slaughterhouse (Hanbo, Korea) approved by Korean government. Pregnant ICR mice were purchased from SAMTACO BIO Inc., Korea. The mice were taken care according to standard protocol of Institute of Laboratory Animal Resources and sacrificed by cervical dislocation after anesthesia.

### Generation and culture of porcine induced pluripotent stem cells (piPSCs)

Pig fetal fibroblasts (PFFs, mixed breed) and mouse embryonic fibroblasts (MEFs) were obtained from approximately 27-day-old and 14-day-old fetuses after artificial insemination, respectively. The head, limbs, and internal organs were removed. The remaining tissue was minced and cultured in DMEM (Welgene, Korea) supplemented with 10% fetal bovine serum (FBS; collected and processed in the United States; Genedepot, TX, USA), 1× glutamax (Gibco), 0.1 mM ß-mercaptoethanol (Gibco), and 1× antibiotic/antimycotic (Gibco). piPSC derivation was conducted using lentiviral vectors with inducible systems containing human OCT4, SOX2, KLF4, and MYC. Lentiviral vector production and transduction were performed as described previously [29]. Five plasmids were used for the production of lentiviral vectors: FUW-tetO-hOCT4, FUW-tetO-hSOX2, FUW-tetO-hKlf4, FUW-tetO-hMYC, and FUW-M2rtTA. Cultured female PFFs were infected with lentiviral vectors for 48 hours. Infected PFFs were transferred onto feeder cells composed of mitotically inactivated MEFs and cultured with reprogramming media for 2 weeks. The reprogramming media contained DMEM (Welgene) supplemented with 15% FBS, 2 mM glutamax, 0.1 mM ß-mercaptoethanol, 1× MEM non-essential amino acids (Gibco), 1× antibiotic/antimycotic, 2 ng/ml doxycycline (dox), and 1000 unit/ml Leukemia inhibitory factor (LIF; Millipore, MA, USA). Two weeks post-infection, primary colonies of piPSCs were stained with AP live stain kit as described below, and AP-positive colonies were selected for further analyses and culture. Established piPSCs were cultured under culture media supplemented with 1000 unit/ml LIF or 1000 unit/ml LIF, 3 μM CHIR99021 (Cayman chemical, MI, USA) and 1 μM PD0325901 (Selleckchem, TX, USA; inhibitors for GSK3 and MEK/ERK respectively; 2i) or 10 ng/ml basic fibroblast growth factor (bFGF; R&D Systems, MN, USA). Media were changed every day and all cells were cultured under humidified conditions with 5% CO_2_ at 37°C. When colonies of piPSCs were grown sufficiently for passaging, cells were subcultured into new feeder cells containing mitomycin-C-treated (Roche, Switzerland) MEFs.

### Embryoid body (EB) formation and in vitro differentiation

To evaluate the *in vitro* differentiation ability, embryoid bodies were generated from piPSCs. Cultured piPSCs were dissociated into single cells using 0.25% trypsin/EDTA solution (Welgene) and cultured in petri dishes without cytokines for 5 days. After suspension culture, dissociated cells were aggregated and formed embryoid bodies. Cultured embryoid bodies were seeded on 0.1% gelatin-coated plates and cultured for 2–3 weeks with DMEM containing 15% FBS. After 2–3 weeks, differentiated cells were fixed with 4% paraformaldehyde and analyzed by immunostaining with the following antibodies: Neurofilament (ectoderm), Vimentin (mesoderm), and Cytokeratin 17 (endoderm), as described below.

### Alkaline phosphatase (AP) staining

We used two alkaline phosphatase (AP) staining kits for staining both fixed and live cells. For the fixed cells, cells were fixed with 4% paraformaldehyde for 30 min. After washing, fixed cells were stained with a solution containing nitro blue tetrazolium chloride (NBT) and 5-bromo-4-chloro-3-indolyl phosphate toluidine salt (BCIP) stock solution (Roche) in a buffer solution for 30 min at room temperature. Cells were then examined under an inverted microscope. AP staining of live cells was performed using the AP live stain kit (Molecular probes, OR, USA) according to the manufacturer’s instructions. The stained cells were examined under a fluorescence microscope and AP-positive colonies were selected for further cell culture and analyses.

### Immunocytochemistry (ICC) analyses

ICC analyses were performed to evaluate the expression of genes related to pluripotency and differentiation. Before staining, all cell samples were preincubated for 10 min at 4°C and fixed with 4% paraformaldehyde for 30 min. After washing twice with Dulbecco’s phosphate-buffered saline (DPBS; Welgene), samples were treated for 1 h with 10% goat serum in DPBS to prevent nonspecific binding. Serum-treated cells were incubated overnight at 4°C with primary antibodies. The primary antibodies used were as follows: OCT4 (Santa Cruz Biotechnology, CA, USA; 1:200), SOX2 (Millipore; 1:200), NANOG (Santa Cruz Biotechnology; 1:200), SSEA1 (Millipore; 1:200), SSEA4 (Millipore; 1:200), Neurofilament (Millipore; 1:100), Vimentin (Millipore; 1:100), and Cytokeratin 17 (Millipore; 1:100). When we used the antibodies for intracellular proteins such as OCT4, SOX2, and NANOG, fixed cells were treated for 5 min with 0.2% Triton-X100 (Sigma-Aldrich, MO, USA) before serum blocking. After incubation with the primary antibody, the cells were treated for 3 h at room temperature with Alexa Fluor-conjugated secondary antibodies. Nuclei were stained with Hoescht 33342 (Molecular Probes). Images of stained cells were captured using a LSM 700 Laser Scanning Microscope (Carl Zeiss, Germany) and processed with the ZEN 2012 Light Edition program (Carl Zeiss).

### Flow cytometric analyses

To verify expression of pluripotent genes in piPSCs cultured in media supplemented with LIF, LIF + 2i, or bFGF, we performed flow cytometric analyses. Dissociated piPSCs were fixed with 4% paraformaldehyde. The fixed cells were permeabilized in 0.1% Triton-X 100 (Sigma Aldrich) for 5 min and then incubated with 10% goat serum (blocking solution). The cells were then incubated with primary antibodies at 4°C overnight. The primary antibodies used were as follows: OCT4 (Santa Cruz Biotechnology, 1:200), SOX2 (Millipore, 1:200), and NANOG (Santa Cruz Biotechnology, 1:200). After incubation with primary antibody, the cells were treated for 3 h at room temperature with Alexa Fluor-conjugated secondary antibodies. The stained cells were analyzed using flow cytometery (FACSCalibur) and Cell Quest software (Becton Dickinson, NJ, USA). The resulting data were processed using FlowJo software (Tree Star Inc., OR, USA).

### Polymerase chain reaction (PCR) amplification

To determine whether viral transgenes were inserted into the genome, gDNA were PCR-amplified with transgene-specific primers listed in S1 Table. Genomic DNA was extracted by the G-spin^™^ Total DNA Extraction Kit (iNtRON, Korea). Amplifications were performed using 2× PCR Master mix solution (iNtRON) containing 1 pmol of each primer set and 10 ng gDNA in a 10 μl reaction volume. PCR reactions were performed in a thermocycler under the following conditions: 94°C for 5 min followed by 40 cycles of denaturation at 95°C for 30 s, annealing for 30 s (annealing temperatures depended on each primer set), and extension at 72°C for 30 s, with a final extension at 72°C for 7 min. Amplified PCR products were visualized using electrophoresis on 1% agarose gel stained with ethidium bromide.

### Quantitative real-time polymerase chain reaction (qPCR)

To verify the gene expression level in piPSCs, we performed qPCR. Total RNA from individual samples was extracted using TRIzol^®^ reagent (Invitrogen, MA, USA) according to the manufacturer’s instructions. Complementary DNA was synthesized using a High-capacity RNA-to-cDNA Kit (Applied Biosystems, CA, USA) according to the manufacturer’s instructions, producing a final volume of 20 μl. Extracted cDNA samples were amplified with DyNAmo HS SYBR Green qPCR Kit (Thermo scientific, MA, USA) containing 1 pmol of each primer set listed in S2 Table in a 10 μl reaction volume. Amplification and detection were conducted using the ABI 7300 Real-Time PCR system (Applied Biosystems) under the following conditions: one cycle of 50°C for 2 min and 95°C for 10 min, followed by 40 cycles of denaturation at 95°C for 15 sec and annealing/extension for 1 min (annealing/extension temperatures depended on each primer set). We analyzed the dissociation curve and loaded the amplified products on gels to confirm the specificity of PCR products. The relative expression level was calculated by normalizing the threshold cycle (Ct) values of each gene to that of the *ACTB* via the Δ^–Ct^ method [30].

### Genome methylation assay

To analyze methylation patterns in porcine OCT4, NANOG, and XIST promoter regions, genomic DNA of piPSCs was analyzed by bisulfite sequencing. Genomic DNA was extracted using the G-spin^™^ Total DNA Extraction Kit (iNtRON) and bisulfite treatment was performed using the EZ DNA Methylation-Gold^™^ Kit (Zymo Research, CA, USA). Bisulfite-treated DNA samples were PCR-amplified with specific primers listed in S3 Table. Amplifications were performed using 2× PCR master mix solution containing 1 pmol of each primer in 20 μl reaction volume. The resulting PCR products were separated by electrophoresis and purified from agarose gels using the MEGAquick-spin^™^ Total Fragment DNA Purification Kit (iNtRON). Purified amplicons were cloned into the pGEMT-Easy Vector (Promega, WI, USA) and transformed into *Escherichia coli* (DH5-α; Novagen, USA). Plasmids were extracted from the selected positive colonies using the DNA-spin^™^ Plasmid DNA Purification Kit (iNtRON). The extracted plasmids were sequenced using an ABI PRISM 3730 automated sequencer (Applied Biosystems). Finally, the methylation patterns of converted sequences with > 95% cytosine-to-thymine conversion rates were analyzed by the BIQ Analyzer Program (http://biq-analyzer.bioinf.mpi-inf.mpg.de/).

### Karyotyping

Karyotyping of cells using standard G-banding chromosome and cytogenetic analysis was performed at GenDix Laboratories (Korea; www.gendix.com).

### Statistical analyses

All gene expression data from qPCR analyses were statistically analyzed using GraphPad Prism 6 statistical software (GraphPad Software, CA, USA). Statistical differences between datasets were determined by one-way analyses of variance (ANOVAs) followed by Fisher’s least significant difference (LSD) tests. Differences were considered significant at P < 0.05.

## Results

### Derivation of piPSCs from PEFs with a drug-mediated inducible gene expression system

**Fig 1:**
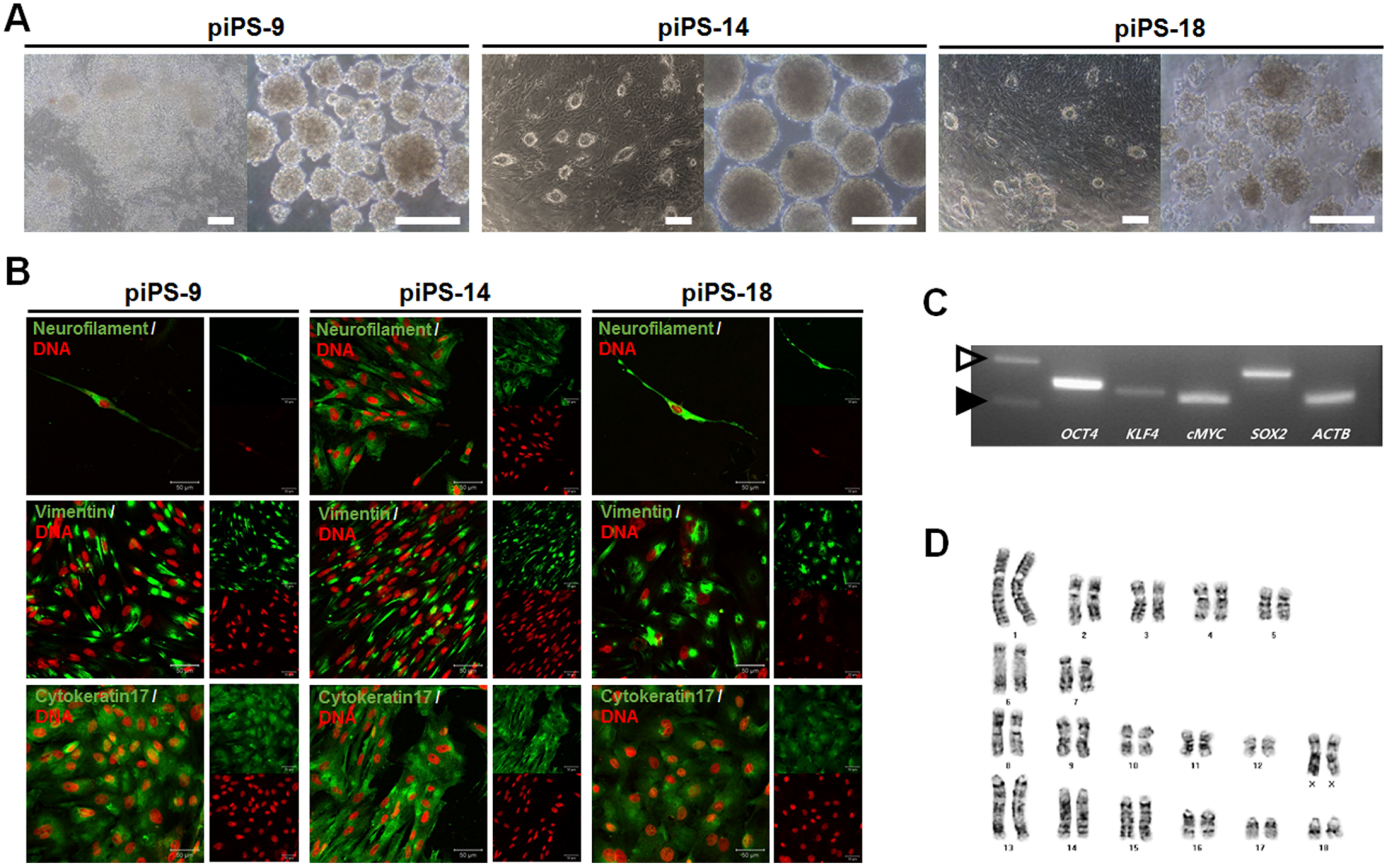
Characterization of three selected iPSC lines. (A) Three cell lines, including one fussy type, piPS-9 and two naïve types, piPS-14 and 18, were selected. When the cells were cultured in suspension, the cells aggregated and subsequently formed embryoid bodies. (B) The formed EBs differentiated into three germ layers after being placed on gelatin-coated plates. Expression of three germ layer markers including Cytokeratin17 (endoderm), Vimentin (mesoderm), and Neurofilament (ectoderm) was confirmed by immunostaining. Of the three cell lines, piPS-14 (showing better morphology and differential potentials with larger EBs than other cell lines) was selected for further analyses and culture. (C) The integration of four transgenes into the genome was confirmed. Black and hollow arrows indicate 100 bp and 200 bp size markers, respectively. (D) The piPS-14 cell line could be stably maintained with a normal karyotype (36 + XX). Scale bar = 200 μm in A; 50 μm in B.

Because gene expression is easily regulated by drugs, drug-inducible vector systems have been used for iPSC studies on the generation of iPSCs, secondary iPS systems [31], reprogramming mechanisms [11, 12], and inducing naïve pluripotent stem cells in humans [2, 3]. Thus, we used a doxycycline-mediated inducible gene expression system for generating pig iPSCs. Two weeks post-infection, we observed several primary colonies (S1A and S1B Fig). Because AP-positive and negative colonies were observed simultaneously, AP-positive colonies were selected using AP live stain kit under a florescence microscope (S1C Fig). Twenty colonies were picked to confirm the integration of four transgenes into the genome (S1D Fig). The 20 confirmed colonies showed various morphologies, including fussy and naïve types, and differing numbers of transgenes. We selected three cell lines (one fussy type, piPS-9, and two naïve types, piPS-14 and 18) for further analyses (Fig 1A). When the cells were cultured in suspension, the cells aggregated and subsequently formed embryoid bodies (Fig 1A). These EBs differentiated into three germ layers when placed on gelatin-coated plates. Expression of three germ layer markers including Cytokeratin17 (endoderm), Vimentin (mesoderm), and Neurofilament (ectoderm) was confirmed by immunostaining (Fig 1B). Interestingly, selected AP-negative colonies possessed no *in vitro* differentiation potential and reverted to fibroblasts with no hSOX transgenes (S2 Fig). Of the three cell lines, piPS-14 showed better morphology and differential potential with larger EBs, and was selected for further analyses and culture. Additionally, we confirmed the integration of four transgenes into the genome (Fig 1C). The piPS-14 cell line could be stably maintained with normal karyotypes for > 50 passages (Fig 1D). Thus, we generated iPSCs with similar *in vitro* differentiation potential and self-renewal to typical pluripotent stem cells.

### The pluripotent state of piPSCs could be modulated by culture conditions

**Fig 2:**
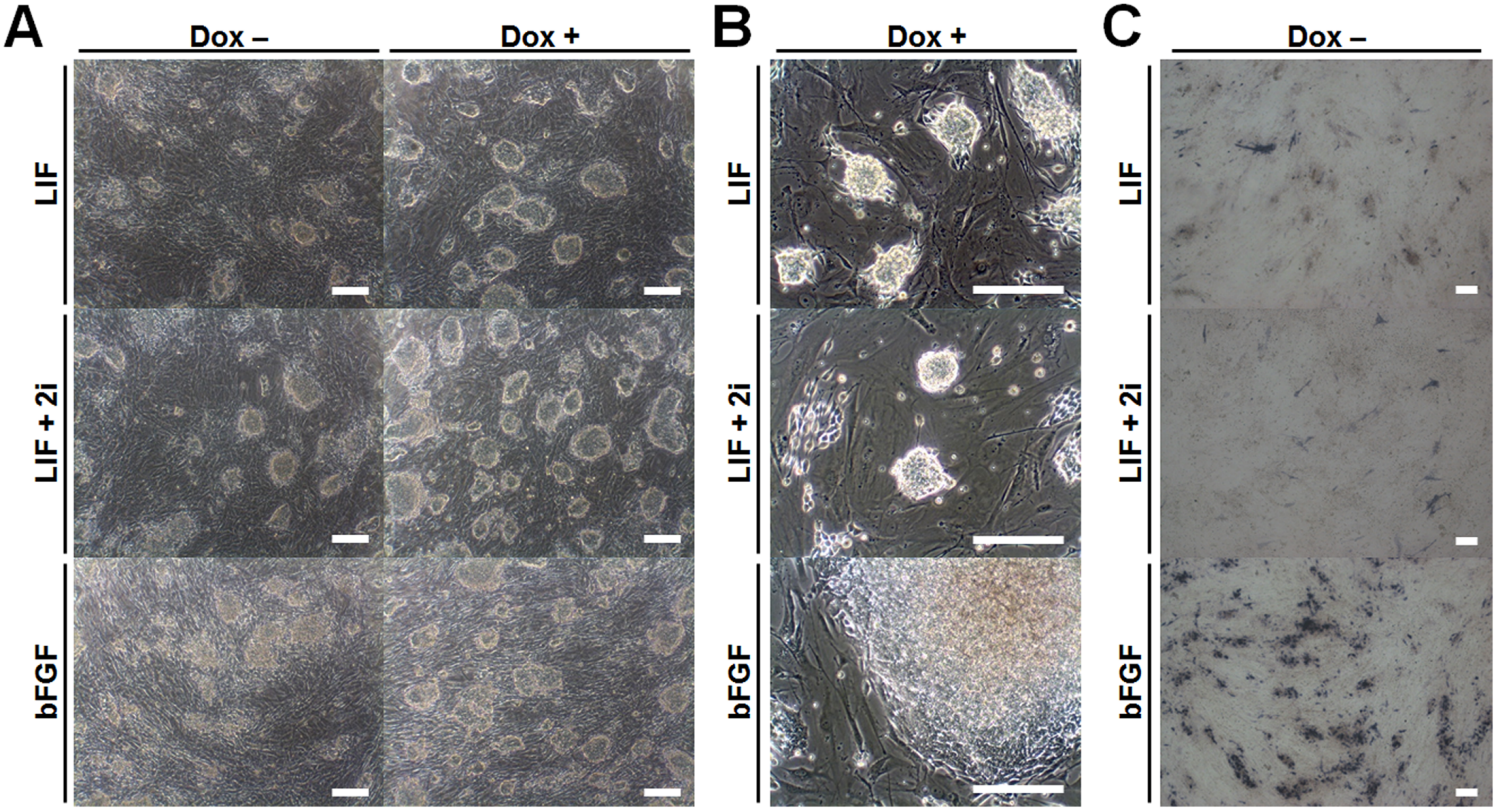
Morphological changes of piPSCs in response to culture conditions. The piPS-14 cells cultured under LIF conditions were transferred to two different media supplemented with LIF + 2i or bFGF. (A) Four days after changing culture media, the cells cultured in bFGF started to change morphologically, becoming flattened. (B) Later, the cells cultured with bFGF showed a primed-like flattened morphology, while those cultured with LIF or LIF + 2i still showed compact dome-shaped morphology. (C) When cultured in the absence of dox, in all cells under the three conditions, the number of colonies gradually decreased within 4 days, and AP-positive colonies were absent after subcultures. Scale bar = 200 μm.

**Fig 3:**
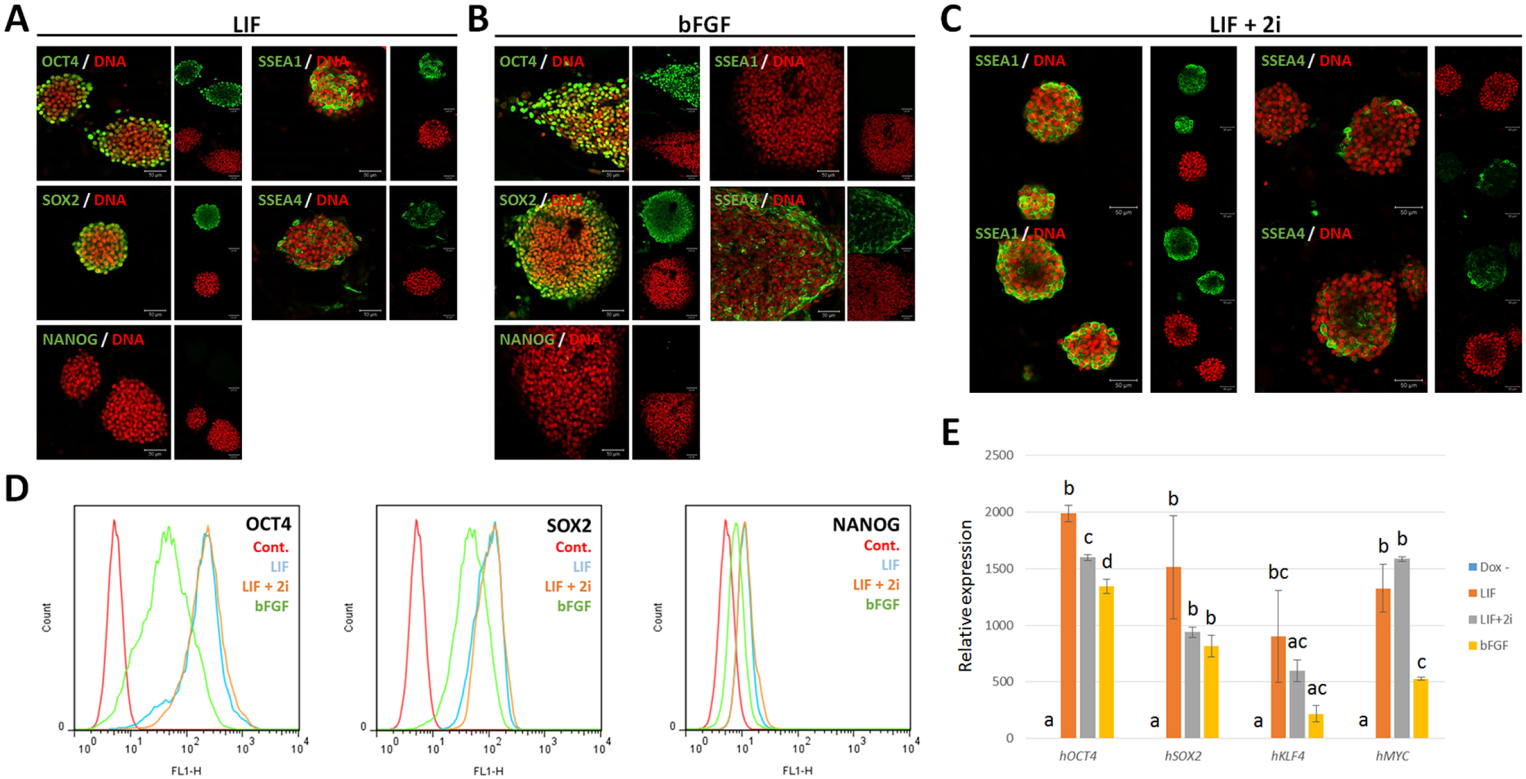
Expression of pluripotent markers in piPSCs. Expression of endogenous and exogenous pluripotent genes was determined by immunostaining and qPCR. (A) OCT4, SOX2, SSEA1, and SSEA4 were expressed in naïve-like piPSCs cultured with LIF. (B) OCT4, SOX2, and SSEA4 were expressed in primed-like piPSCs cultured with bFGF (C) When treated with 2i, SSEA4 was still expressed in naïve-like piPSCs. (D) Expression of NANOG was not detected under any culture conditions as determined by flow cytometric analysis. (E) Exogenous transgenes were highly expressed when treated with doxycycline in piPSCs, while transgenes were not expressed in the absence of doxycycline. Scale bar = 50 μm.

The generated piPS-14 cell line under LIF-supplemented conditions showed a naïve-like pluripotent state with short term subculture (3 days), single cell colonization, and dome-shaped morphology. We explored whether the pluripotent state of piPSCs could be modulated based on culture conditions. The piPS-14 cells cultured in LIF conditions were transferred to two different media supplemented with LIF + 2i or bFGF. Four days after changing the culture media, the cells cultured in bFGF started to change morphologically, becoming flattened (Fig 2A). After extended culturing, the cells cultured with bFGF showed a primed-like flattened morphology, while cells cultured with LIF or LIF + 2i still showed compact dome-shaped morphology (Fig 2B). In addition, when cultured in the absence of dox, the number of colonies gradually decreased within 4 days for cells under all three conditions, and AP-positive colonies were absent after subcultures (Fig 2C). Immunostaining analyses revealed two representative results based on gene expression patterns. First, NANOG expression was not detected under any conditions. NANOG, a key pluripotent gene, is reactivated during the late stages of reprogramming [12, 32]. However, no cells under any culture conditions expressed NANOG, though OCT4 and SOX2 were highly expressed, as determined by ICC and flow cytometric analyses (Fig 3A, 3B and 3D). Second, pluripotent markers such as SSEA1 and SSEA4 were differentially expressed depending on culture conditions (Fig 3A and 3B). According to previous studies, SSEA1 and SSEA4 are specific markers for the naïve and primed pluripotent state, respectively [1]. Consistent with previous studies, SSEA1 and SSEA4 were exclusively detected in cells cultured with LIF and bFGF, respectively. Notably, in naïve-like cells, SSEA1 expression patterns were heterogeneous within colonies, and parts of the cells still expressed SSEA4 as a primed-pluripotent marker (Fig 3A). It is possible that bFGF from feeder cells could affect SSEA4 expression under LIF-supplemented conditions. When treated with 2i to enhance the naïve state by inhibiting ERK/MEK and GSK signaling, although SSEA1 was expressed homogenously, SSEA4 was still expressed in specific cell regions (Fig 3C). When injected into immunodeficient mice to verify potential for *in vivo* differentiation, no cultured cell lines could form teratomas (data not shown). These results indicate that the pluripotent state of derived piPSCs can be modulated by manipulating culture conditions, but that these cells may be partially reprogrammed and depend on the transgenes.

### Endogenous genes related to pluripotency were not sufficiently reactivated by exogenous factors

**Fig 4:**
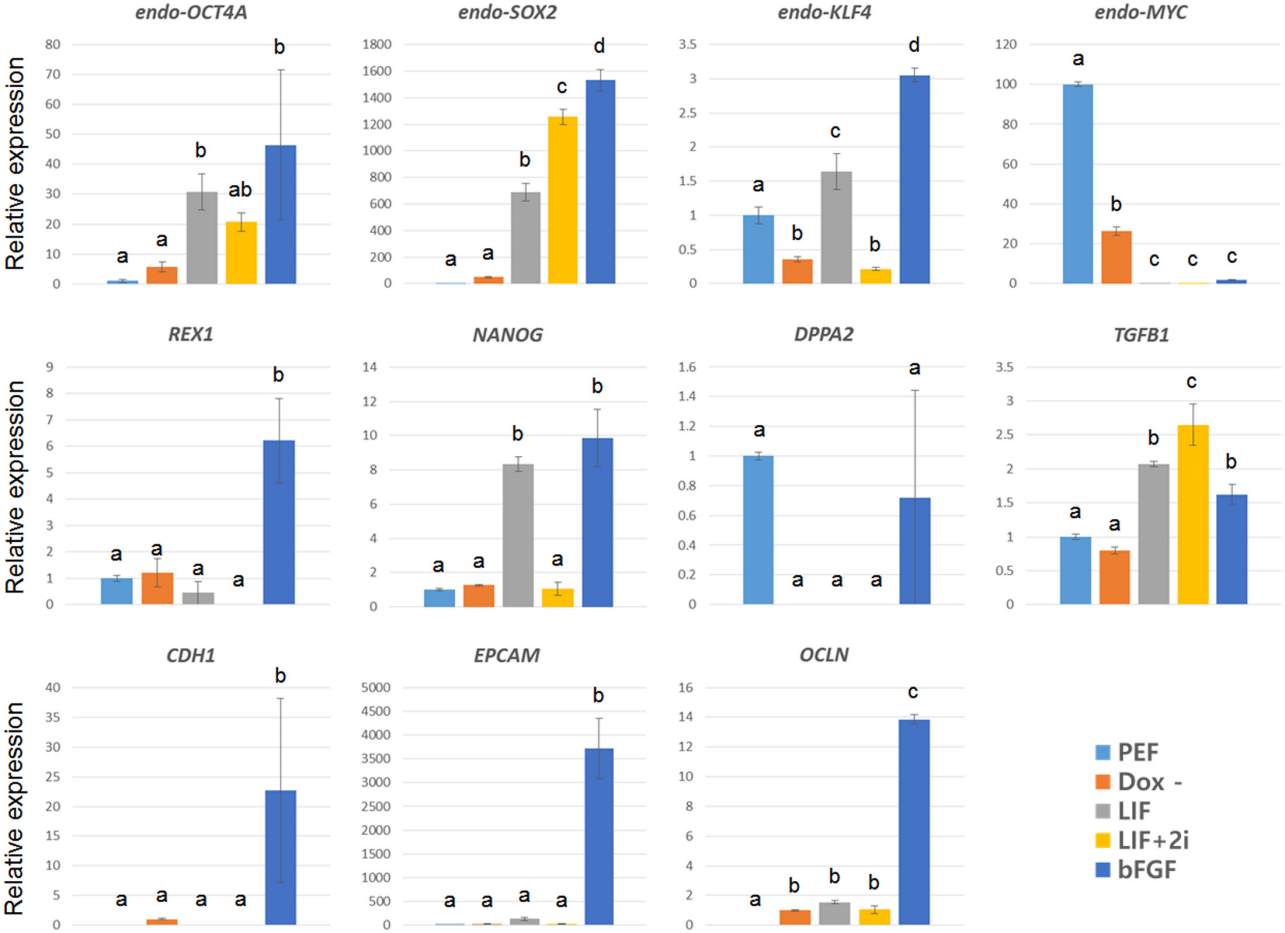
Expression of pluripotent and MET-related genes as measured by qPCR. To verify the effects of transgenes on endogenous genes, expression levels of endogenous genes related to pluripotency and MET (pluripotent genes: *pOCT4a*, *pSOX2*, *pKLF4*, *pMYC*, *NANOG*, *DPPA2* and *REX1*; epithelial-to-mesenchymal transition (EMT) inducer: *TGFB1*; epithelial-specific markers: *CDH1*, *EPCAM* and *OCLN*) were determined by qPCR. As reprogramming is processed by exogenous genes, endogenous pluripotent genes (*pOCT4a*, *pSOX2*, *pKLF4*, *NANOG*, and *REX1*) and epithelial-specific markers (*CDH1*, *EPCAM*, and *OCLN*) were upregulated. However, *pMYC* and *DPPA2* (known predictors of reprogramming) were not reactivated, while *TGFB1* (blocks reprogramming) was not efficiently shut-down by transgene expression. Some genes, such as *pSOX2*, *pKLF4*, *REX1*, and epithelial-specific markers, were preferentially expressed in culture conditions containing bFGF, and *pKLF4* and *NANOG* were downregulated when treated with 2i.

Based on the above results, generated piPSCs were incompletely reprogrammed and depended on transgenes. For complete reprogramming, endogenous pluripotent genes must be reactivated without transgene expression [13, 14]. During the reprogramming process from fibroblasts to iPSCs, several events occur, including mesenchymal-to-epithelial transition (MET) [10] and reactivation of endogenous pluripotent genes [11]. To verify the effects of transgenes on endogenous genes, expression levels of endogenous genes associated with pluripotency and MET (pluripotent genes: pig (p) *OCT4a*, *pSOX2*, *pKLF4*, *pMYC*, *NANOG*, *DPPA2*, and *REX1;* epithelial-to-mesenchymal transition (EMT) inducer: *TGFB1*; epithelial-specific markers: *CDH1*, *EPCAM*, and *OCLN*) were determined by qPCR (Fig 4). In infected cells, exogenous transgenes were highly expressed when treated with doxycycline, while transgenes were not expressed in the absence of doxycycline (Fig 3E). As reprogramming was processed by exogenous genes, endogenous pluripotent genes (*pOCT4a*, *pSOX2*, *pKLF4*, *NANOG*, and *REX1*) and epithelial-specific markers (*CDH1*, *EPCAM*, and *OCLN*) were upregulated, as reported previously [10, 11]. However, *pMYC* and *DPPA2*, known predictors of reprogramming, were not reactivated, while *TGFB1* blocked reprogramming and was not efficiently shut down by transgene expression. Thus, although exogenous *MYC* was expressed as determined by RT-qPCR (Fig 3E), lack of endogenous MYC (a repressor of TGFB1 during MET) [10] led to insufficient downregulation of *TGFB1*. Interestingly, some genes, such as p*SOX2*, *pKLF4*, *REX1*, and epithelial-specific markers, that were preferentially expressed under culture conditions containing bFGF, and *pKLF4* and *NANOG*, were downregulated when treated with 2i. Taken together, these data demonstrate that piPSCs were incompletely reprogrammed because endogenous genes were insufficiently reactivated by transgene expression.

### Bisulfite sequencing showed that piPSCs were not epigenetically reprogrammed

**Fig 5:**
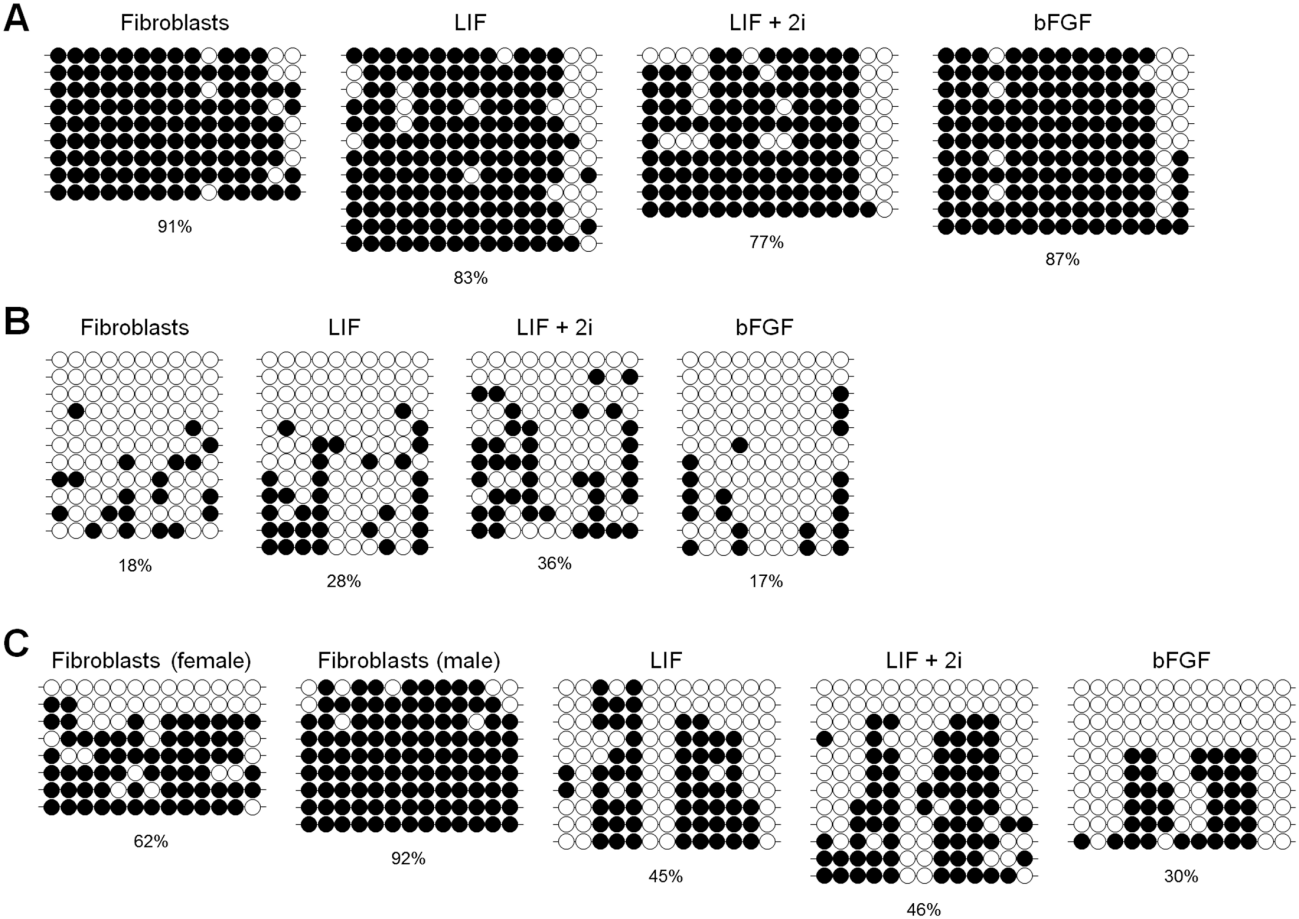
Bisulfite sequencing at promoter regions of OCT4, NANOG, and XIST. To verify whether epigenetic reprogramming occurred by expression of exogenous genes, DNA methylation patterns at promoter regions of pig OCT4, NANOG, and XIST were evaluated by bisulfite sequencing. (A) OCT core promoter: OCT4 core promoter regions were highly methylated. (B) NANOG promoter: Promoter regions of NANOG were methylated to levels similar to somatic cell control. (C) XIST promoter: X chromosome reactivation did not occur in naïve-like piPSCs. Each circle indicates individual CpG dinucleotides. White and dark circles represent unmethylated and methylated CpGs, respectively. Each row represents one individual clone of amplified PCR products.

The piPSCs were partially reprogrammed as determined by qPCR and immunostaining. To confirm whether epigenetic reprogramming occurred due to the expression of exogenous genes, DNA methylation patterns at promoter regions of pig OCT4, NANOG, and XIST were evaluated using bisulfite sequencing. Although endogenous OCT4 (a key factor in pluripotency) [33] was expressed in piPSCs as detected by qPCR, OCT4 core promoter regions were highly methylated (Fig 5A). In the late stages of reprogramming, X chromosome reactivation occurred as NANOG expression was elevated in naïve pluripotent stem cells [3, 32, 34]. However, in piPSCs, NANOG promoter regions were methylated at levels similar to somatic cell control (Fig 5B), and X chromosome reactivation did not occur in naïve-like piPSCs (Fig 5C). As shown in Figs 3 and 4, although *NANOG* expression was detected at the mRNA level, it was not detected at the protein level as determined by immunostaining. Consistent with these results, *DPPA2* (a pluripotent gene interacting with NANOG; [11, 35] was not expressed in piPSCs (Fig 4). These data indicate that transcripted *NANOG* mRNA can be not translated or that the amount of translated NANOG protein may be too low to affect reprogramming. Similar phenomenon was observed in pre-implantation pig embryos, which indicates that post-transcriptional mechanisms regulate NANOG expression [36]. Bisulfite sequencing combined with gene expression data showed that epigenetic reprogramming of piPSCs did not occur with incomplete reactivation of endogenous genes.

## Discussion

### Pluripotent stem cells of pigs as a non-permissive species

Recently, several studies have suggested that the states of PSCs are divided into two categories: naïve and primed. Naïve PSCs derived from early epiblasts in pre-implantation blastocysts as a developmental ground state can generate the chimeric fetus when micro-injected into recipient blastocysts. In addition, primed PSCs derived from late epiblasts in post-implantation blastocyst possess more differentiated pluripotency than naïve cells in terms of developmental capacity, gene expression, and epigenetic signatures. In permissive lines, both pluripotent states of PSCs can be derived from embryos. However, in nonpermissive lines, the stem cells cannot be stabilized in the naïve state, and are instead differentiated and stabilized at the primed state during the establishment process if no additional treatments (including genetic manipulation and chemicals) are performed [1, 37]. It has been suggested that, as a nonpermissive species, pig epiblasts are reprogrammed into primed state cells during the establishment of PSCs from embryos [38]. In addition, piPSCs derived from porcine somatic cells are morphologically and molecularly similar to mEpiSCs or hESCs, rather than to mESCs [22, 23, 38–42].

Several studies have attempted to establish naïve-state pluripotent stem cells from non-permissive species such as rats and humans [43, 44]. The first case of human naïve PSCs reported that derivation of naïve PSCs were accomplished via ectopic expression of OCT4 and KLF4 supplemented with LIF and two inhibitors for GSK and ERK1/2 signaling [3]. However, these cell lines could not be maintained without transgene expression. Recent studies have reported that generation of transgene-free human naïve-like PSCs could be achieved by several small molecules in addition to 2i [45–50]. These results demonstrated that modulating signaling pathways is required for the maintenance of naïve state pluripotent stem cells in non-permissive species.

Consistent with previous studies involving pig ESCs and iPSCs, our data show that pig is a non-permissive species. During reprogramming, bFGF treatment more strongly upregulated specific pluripotent genes such as p*SOX2*, *pKLF4*, *REX1*, and epithelial-specific markers compared to LIF treatment, and blocking bFGF signaling downregulated *pKLF4* and *NANOG* rather than completing reprogramming, as reported in mice (Fig 4)[21]. Furthermore, although SSEA1 and SSEA4 were exclusively expressed in LIF- and bFGF- supplemented conditions, respectively, specific cell regions cultured with LIF expressed SSEA4 (Fig 3A and 3B). Inhibiting ERK/MEK and GSK signaling did not block differentiation of piPSCs from the naïve state into the primed state (Fig 3C). Overall, pig is less permissive of generating naïve pluripotency than human, because pig iPSCs are ready to enter a primed state when cultured with 2i, while human ESCs could be converted into the naïve state by LIF, 2i, and transgene expression [3]. Therefore, as a non-permissive species, pig somatic cells are preferentially reprogrammed into the primed state depending on bFGF signaling.

### Incomplete reprogramming was induced by the failure of endogenous gene reactivation

To improve the efficiency of nuclear reprogramming from somatic cells to pluripotent cells, reprogramming mechanisms and gene expression patterns have been elucidated in previous reports. At the early stage of reprogramming, mesenchymal-to-epithelial transition (MET) occurred, which upregulated epithelial-specific markers and downregulated Tgfb1 (which blocks nuclear reprogramming) by expressing Yamanaka’s factors [10, 51]. The cells that convert to iPSCs express predictive markers, such as Esrrb, Utf1, Lin28, and Dppa2. In the late stage, reprogramming is completed with epigenetic remodeling by upregulating endogenous genes via Sox2 and reactivating X chromosomes (in the case of female cells) via Nanog [11, 12]. Simultaneously, full reprogramming of the pluripotent state requires the activation of endogenous pluripotency genes along with the silencing of transgenes for stable maintenance of the pluripotent state. [13, 14]. Because the defective silencing of transgenes after reprogramming affects stability, carcinogenesis, and differentiation ability of iPSCs, silencing or eliminating transgene expression is one of the most important step for fully reprogrammed iPSCs [20, 52].

It has been reported that incompletely reprogrammed iPSCs resulted in defective reprogramming processes including insufficient downregulation of Tgfb1, lack of Nanog expression, and failure of transgene silencing. Compared with pre-iPSC lines, several common characteristics, such as incomplete expression of pluripotent genes, inactive X chromosomes in female cells, and inability to generate germline chimeras, have been observed [10, 20, 21, 32]. In porcine studies, the majority of pig iPSC lines were partially reprogrammed, showing dependence on transgenes, lack of NANOG expression, failure of epigenetic reprogramming, and inability to form chimeras [22–27]. According to previous studies, the majority of piPSC lines exhibited dependency of continuous transgene expression, and it was impossible to obtain transgene-independent colonies using a transgene-free system such as drug-inducible, episomal or plasmid vectors [23, 26, 39, 53]. In recent study using cells carrying OCT4-tdTomato reporter, no dtTomato expression was detected in pig iPSCs induced by human Yamanaka’s factors [54].

Since Nanog as a gateway to pluripotency plays pivotal roles in epigenetic remodeling and X chromosome reactivation, it is important to reactivate Nanog in the late stages of reprogramming in mouse [32, 55]. In human as a non-permissive species, NANOG plays an important role in reprogramming and maintaining pluripotency [56, 57]. In some pig studies, NANOG upregulation and epigenetic reprogramming were not achieved [26, 27]. Consistent with previous studies, we confirmed that generated iPSCs carrying whole Yamanaka’s factors have no NANOG expression, and that epigenetic alteration relied on the expression of transgenes for maintenance (Figs 3C and 5). Although Nanog could be reactivated by treatment of 2i in mouse pre-iPSCs of permissive species [21], *NANOG* mRNA expression level could not be upregulated by 2i treatment in our pig iPSCs (Fig 4). Furthermore, insufficient downregulation of *TGFB1* resulted in abnormal MET. In addition, during the reprogramming process, we obtained AP-negative cell lines, as reported previously [8, 24, 27]. These iPSC lines lacking AP activity did not express SOX2 without integration of exogenous SOX2 (S2 Fig). It is possible that SOX2 expression is correlated with AP activity. Taken together, Yamanaka’s factors may be not sufficient to achieve MET and epigenetic resetting in pig. It is important to identify optimal culture conditions and novel factors for the reprogramming of pig somatic cells.

### New approaches are required to overcome reprogramming hurdles and generate pig iPSCs

To overcome the barriers of reprogramming described above, various studies have been performed using small molecules, another reprogramming factor, and nutrient supplements. At this time, drug-inducible vector systems, which can easily turn transgenes on and off, have been used in various studies involving the iPSC generation and the elucidation of reprogramming mechanisms [11, 12, 31]. Chromatin remodeling and the erasing of epigenetic memory in somatic cells have been accomplished by inhibiting DNA methylation and using chromatin modifiers [17]. Pre-iPSCs lacking Nanog expression were converted into fully reprogrammed iPSCs by inhibiting bFGF signaling and Nanog overexpression [21, 58]. In addition, serum free media and vitamin C could be used to overcome hurdles and increase reprogramming efficiency [59, 60]. In addition, suppression of Tgfb signaling, apoptosis, senescence, and cell-cycle arrest are considered useful tools for reducing reprogramming barriers [18, 19, 61, 62]. Recently, it has been shown that several factors including Nanog, Lin28, Nr5a2, and Glis1 could be used for nuclear reprogramming instead of Yamanaka’s factors [56, 63, 64]. Signaling inhibitors that elevate pluripotent status to the naïve state have been revealed in human studies [45, 47, 50]. Accordingly, if various tools are properly utilized, it may be possible to generate fully-reprogrammed porcine iPSCs by overcoming the genetic and epigenetic barriers.

However, it is challenging to establish porcine ESCs and optimize pig-specific reprogramming conditions using typical methods because of differences in the genetic backgrounds of mouse, human, and pig. During development of the early embryo, which has an inner cell mass (ICM) considered to be the pluripotent cell population, pig has a prolonged preimplantation period compared with mouse and human [28]. Therefore, in pig embryos, different cell signaling that governs pluripotency reveals differences compared to mouse embryos. Unlike mouse, in which Oct4 and Cdx2 are exclusively expressed in ICM and trophectoderm (TE), respectively, in pig, SOX2 is specifically expressed in ICM while OCT4 is expressed in TE until the blastocyst expands on embryonic day 8 [65]. In ICM and epiblasts of pig blastocysts, the LIF receptor is absent while FGF receptors are specifically expressed, which indicates that FGF signaling may play an important role in the maintenance of pluripotency rather than LIF [66]. Along with FGF signaling, BMP signaling plays an important role in maintaining pluripotency [36].

Recent studies have increased our understanding of pig somatic cell reprogramming. First, pig iPSCs could be induced by KLF4, SOX2, and MYC (but not OCT4) infection, although transgenes were not silenced [42]. According to that study, the ectopic expression of OCT4 interfered with reactivation of endogenous pluripotent networks in pig fibroblasts, which indicated that OCT4 may not be essential for maintaining pluripotency in pig. However it was reported that endogenous gene expression including OCT4, NANOG and MYC were repressed by expression of counterpart exogenous genes [24]. Similar phenomenon was also occurred in our study (Fig 4). Endogenous MYC was down-regulated as exogenous MYC was activated. These studies suggest that further investigation about reprogramming mechanisms and new reprogramming factors or vector systems are required in pig. Another study showed that generation of piPSCs in which transgenes were silenced was accomplished using combined supplements of three cytokines (LIF, bFGF, and BMP4) and two signaling inhibitors (TGFB1 and WNT inhibitors) [67]. This novel culture condition generated ‘intermediate’ pluripotent stem cells, showing mixed features of naïve and primed states with inactivation of retroviral transgenes. Taken together, it is possible that pig has unique pluripotent characteristics governed by different cell signaling networks than mouse and human. Accordingly, in-depth analyses of pig-specific signaling pathways are required for establishing authentic porcine embryonic stem cells and obtaining completely reprogrammed iPSCs.

## Conclusions

Based on our results, pig iPSCs were stably maintained over an extended period, and could differntiate into three germ layers. In addition, the pluripotent state of iPSCs was regulated by the culture condition. The iPSCs showed a naive- or primed-like pluripotent state in LIF or bFGF supplemented culture conditions, respectively. However, iPSCs were not fully reprogrammed and showed transgene dependency. The cultured iPSCs expressed endogenous transcription factors such as OCT4 and SOX2, but not NANOG (a gateway to complete reprogramming). In addition, endogenous genes related to mesenchymal-to-epithelial transition (*DPPA2*, *CDH1*, *EPCAM*, and *OCLN*) were not sufficiently reactivated and failed to downregulate *TGFB1*. DNA methylation analysis for promoters of OCT4, NANOG, and XIST showed that epigenetic reprogramming did not occur in female iPSCs. Therefore, we showed that Yamanaka’s factors *per se* with typical culture conditions may not be sufficient to achieve MET and epigenetic resetting in pig. Finally, we suggest that in-depth analyses of pig-specific signaling pathways are required to establish authentic porcine embryonic stem cells and obtain completely reprogrammed iPSCs.

## Supporting Information

S1 Fig Generation of pig induced pluripotent stem cells using drug-inducible vectors. A doxycycline-mediated inducible gene expression system was used for generating pig iPSCs. (A, B) Two weeks after infection, several primary colonies were observed. (C) Because AP-positive and negative colonies were simultaneously observed, AP-positive colonies were selected using AP live stain kit under a florescence microscope. (D) Twenty colonies were picked and confirmed the integration of four transgenes into genome. For further analyses, three cell lines were selected. The selected cell lines are indicated by a red box. Scale bar = 200 μm
(TIF)

S2 Fig Characterization of the AP-negative cell line. (A) AP-negative cells have similar morphologies to AP-positive cell lines. (B) When cultured in suspension, the cells could form embryoid bodies. (C, D) However, the cells could not differentiate into three germ layers; only to mesodermal fibroblast-like cells, as determined by immunostaining. (E) hSOX2 was not integrated into the genome of cells. Scale bar = 200 μm in A, B and C; 50 μm in D.
(TIF)

S1 Table Primers for the detection of transgene insertion in gDNA. (DOC)

S2 Table Primers for qPCR. (DOC)

S3 Table Primers for bisulfite sequencing. (DOC)
