# Supplementary figures and images for: Reactivation of Endogenous Genes and Epigenetic Remodeling Are Barriers for Generating Transgene-Free Induced Pluripotent Stem Cells in Pig
Source: PLoS One. 2016 Jun 23;11(6):e0158046. doi: 10.1371/journal.pone.0158046 (PMC4918974; doi:10.1371/journal.pone.0158046)

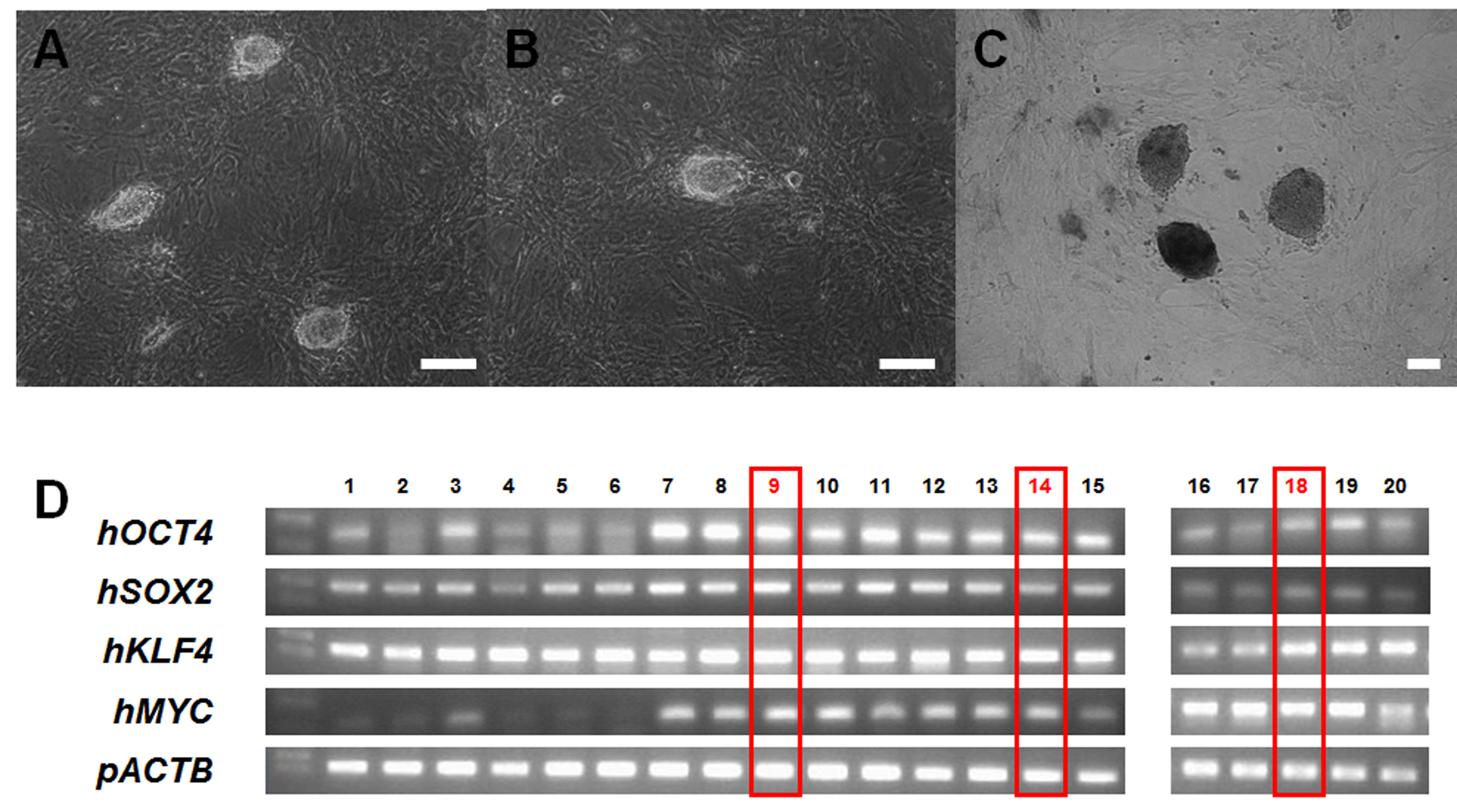

Supplement: S1 Fig — A doxycycline-mediated inducible gene expression system was used for generating pig iPSCs. (A, B) Two weeks after infection, several primary colonies were observed. (C) Because AP-positive and negative colonies were simultaneously observed, AP-positive colonies were selected using AP live stain kit under a florescence microscope. (D) Twenty colonies were picked and confirmed the integration of four transgenes into genome. For further analyses, three cell lines were selected. The selected cell lines are indicated by a red box. Scale bar = 200 μm (TIF) [file pone.0158046.s001.tif]

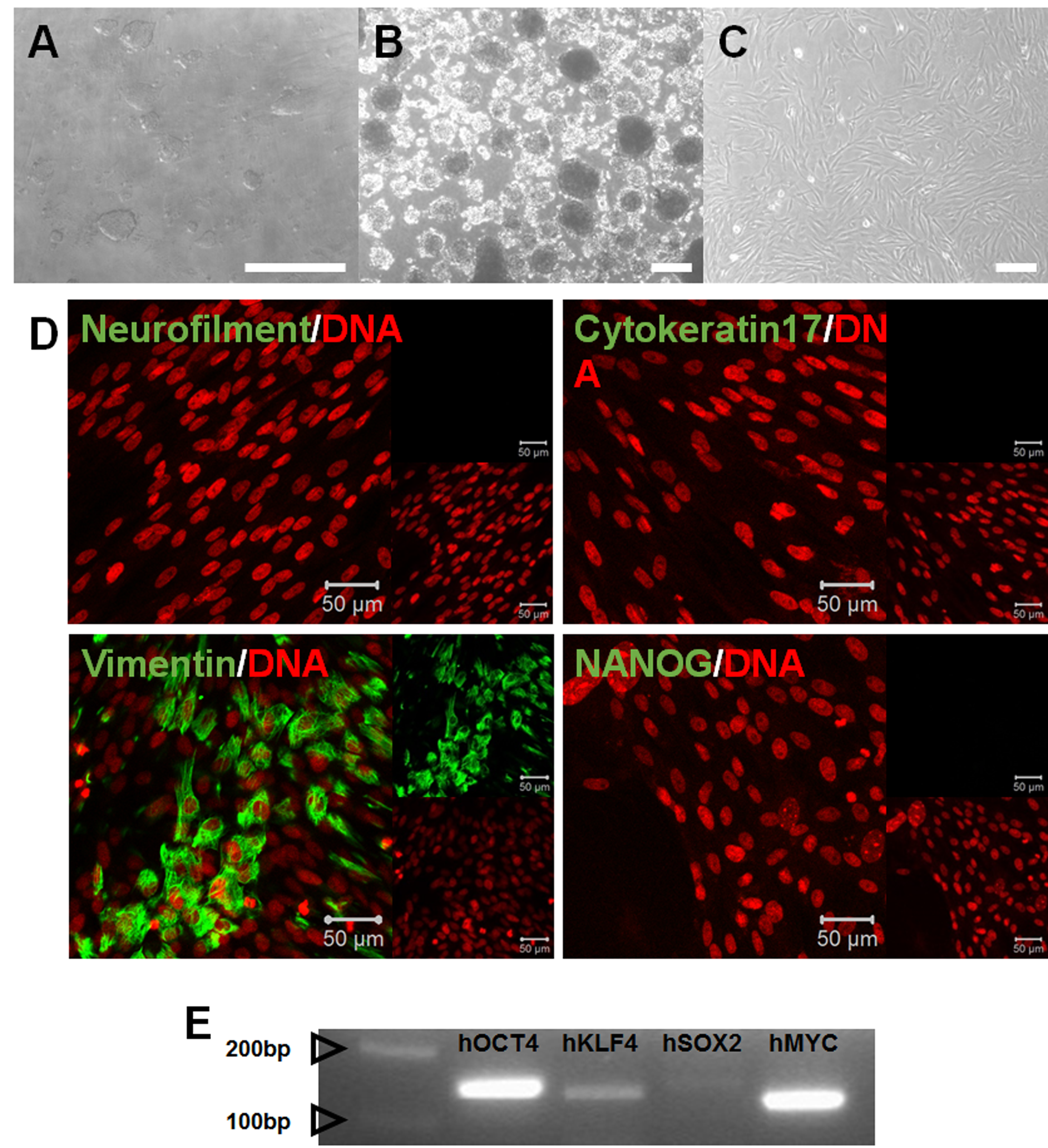

Supplement: S2 Fig — (A) AP-negative cells have similar morphologies to AP-positive cell lines. (B) When cultured in suspension, the cells could form embryoid bodies. (C, D) However, the cells could not differentiate into three germ layers; only to mesodermal fibroblast-like cells, as determined by immunostaining. (E) hSOX2 was not integrated into the genome of cells. Scale bar = 200 μm in A, B and C; 50 μm in D. (TIF) [file pone.0158046.s002.tif]
