# Supplementary material for: Reactivation of Endogenous Genes and Epigenetic Remodeling Are Barriers for Generating Transgene-Free Induced Pluripotent Stem Cells in Pig
Source: PLoS One. 2016 Jun 23;11(6):e0158046. doi: 10.1371/journal.pone.0158046 (PMC4918974; doi:10.1371/journal.pone.0158046)
Supplement: S1 Table — (DOC) [file pone.0158046.s003.doc]

**S1 Table. Primers for the detection of transgene insertion in gDNA.**

| **Gene** | | **Primer sequence** | **PCR product size (bp)** | **Annealing temp (℃)** |
| --- | --- | --- | --- | --- |
| **FUW-tetO-human factors** | *hOCT4* | 5’- CCCCTGTCTCTGTCACCACT -3' | 148 | 60 |
|  |  | 5’- CCACATAGCGTAAAAGGAGCA -3' |  |  |
|  | *hSOX2* | 5’- ACTTCACATGTCCCAGCACT -3' | 180 | 54 |
|  |  | 5’- CATAGCGTAAAAGGAGCAACAT -3' |  |  |
|  | *hKLF4* | 5’- GACCACCTCGCCTTACACAT -3' | 137 | 60 |
|  |  | 5’- CCACATAGCGTAAAAGGAGCA -3' |  |  |
|  | *hMYC* | 5’- CAGCTACGGAACTCTTGTGC -3' | 125 | 60 |
|  |  | 5’- CCACATAGCGTAAAAGGAGCA -3' |  |  |
| **Reference genes** | *ACTB* | 5’- CCGGGACCTGACCGACTACC -3' | 126 | 60 |
|  |  | 5’- TCGAAGTCCAGGGCGACGTA -3' |  |  |
