# Supplementary material for: Reactivation of Endogenous Genes and Epigenetic Remodeling Are Barriers for Generating Transgene-Free Induced Pluripotent Stem Cells in Pig
Source: PLoS One. 2016 Jun 23;11(6):e0158046. doi: 10.1371/journal.pone.0158046 (PMC4918974; doi:10.1371/journal.pone.0158046)
Supplement: S2 Table — (DOC) [file pone.0158046.s004.doc]

**S2 Table. Primers for qPCR.**

| **Gene** | | **Primer sequence** | **PCR product size (bp)** | **Annealing temp (℃)** | **Accession number** |
| --- | --- | --- | --- | --- | --- |
| **Exogenous genes** | *hOCT4* | 5’- CCCCTGTCTCTGTCACCACT -3' | 148 | 60 | FUW-tetO-hOCT4 |
|  |  | 5’- CCACATAGCGTAAAAGGAGCA -3' |  |  |  |
|  | *hSOX2* | 5’- CATTAACGGCACACTGCCC -3' | 132 | 60 | FUW-tetO-hSOX2 |
|  |  | 5’- CATAGCGTAAAAGGAGCAACAT -3' |  |  |  |
|  | *hKLF4* | 5’- GACCACCTCGCCTTACACAT -3' | 137 | 60 | FUW-tetO-hKLF4 |
|  |  | 5’- CCACATAGCGTAAAAGGAGCA -3' |  |  |  |
|  | *hMYC* | 5’- CAGCTACGGAACTCTTGTGC -3' | 125 | 60 | FUW-tetO-hMYC |
|  |  | 5’- CCACATAGCGTAAAAGGAGCA -3' |  |  |  |
| **Endogenous genes** | *OCT4a* | 5’- CTTGGAGAGCCCTGGTTTTACT -3' | 159 | 64 | NM_001113060.1 |
|  |  | 5’- GCCAGGTCCGAGGATCAAC -3' |  |  |  |
|  | *SOX2* | 5’- CGGCGGTGGCAACTCTAC -3' | 100 | 64 | NP_001116669.1 |
|  |  | 5’- TCGGGACCACACCATGAAAG -3' |  |  |  |
|  | *KLF4* | 5’- GGACCACCTTGCCTTACACA -3' | 146 | 60 | NM_001031782.2 |
|  |  | 5’- CTTTCCAGCTGGGTTCCTCC -3' |  |  |  |
|  | *MYC* | 5’- GAAAAAGACGTGCTGCGGAA -3' | 253 | 60 | NM_001005154.1 |
|  |  | 5’- CCAGCCAAGGTTGTGAGGTT -3' |  |  |  |
|  | *NANOG* | 5’- CATCTGCTGAGACCCTCGAC -3' | 195 | 60 | EF_522119.1 |
|  |  | 5’- GGGTCTGCGAGAACACAGTT -3' |  |  |  |
|  | *REX1* | 5’- TCTGAACCCCTCGTGGAAGA -3' | 100 | 60 | XM_005672654.1 |
|  |  | 5’- AGCTTGCTGTAAGCACCTGT -3' |  |  |  |
|  | *TGFB1* | 5’- CGTGCTAATGGTGGAAAGCG -3' | 122 | 60 | NM_214015.1 |
|  |  | 5’- AGAGCAATACAGGTTCCGGC -3' |  |  |  |
|  | *CDH1* | 5’- ATTCTGGGAGGCATCCTTGC -3' | 117 | 64 | NM_001163060.1 |
|  |  | 5’- GTTGTCCCGGGTGTCATCTT -3' |  |  |  |
|  | *EPCAM* | 5’- TGCTCTTTGAATGCGCTTGG -3' | 172 | 60 | NM_214419.1 |
|  |  | 5’- AGAGCCCATCGTTGTTCTGG -3' |  |  |  |
|  | *OCLN* | 5’- CAGTGGTAACTTGGAGGCGT -3' | 104 | 60 | NM_001163647.2 |
|  |  | 5’- CCGTCGTGTAGTCTGTCTCG -3' |  |  |  |
|  | *DPPA2* | 5’- CTTCAAGAGCCGTTCACCCT -3' | 144 | 60 | XM_003358822.2 |
|  |  | 5’- GGCGAACCCAACCTTCTGTA -3' |  |  |  |
| **Reference gene** | *ACTB* | 5’- GTGGACATCAGGAAGGACCTCTA -3' | 131 | 64 | XM_003357928.2 |
|  |  | 5’- ATGATCTTGATCTTCATGGTGCT -3' |  |  |  |
