# Supplementary material for: Reactivation of Endogenous Genes and Epigenetic Remodeling Are Barriers for Generating Transgene-Free Induced Pluripotent Stem Cells in Pig
Source: PLoS One. 2016 Jun 23;11(6):e0158046. doi: 10.1371/journal.pone.0158046 (PMC4918974; doi:10.1371/journal.pone.0158046)
Supplement: S3 Table — (DOC) [file pone.0158046.s005.doc]

**S3 Table. Primers for bisulfite sequencing.**

| **Gene** | | **Primer sequence** | **PCR product size (bp)** | **Annealing Tm (℃)** | **CpG** | **Reference** |
| --- | --- | --- | --- | --- | --- | --- |
| ***OCT4*** | | 5’- GAAGAGGGGTTTAATATTTGGTTTT -3' | 281 | 58 | 15 |  |
|  |  | 5’- CCACCCACTAACCTTAACCTCTAA -3' |  |  |  |  |
| ***NANOG*** | | 5’- GGAGATTTAAAGGAGTTTTAGGTTAAGAAA -3' | 500 | 58 | 10 |  |
|  |  | 5’- TCTCCTCCAAATATTAAAAATATCAAAAA -3' |  |  |  |  |
| ***XIST*** | outer | 5’- TGGTTAAATGAGGTATTTGGA -3' | 525 | 54 | 13 |  |
|  |  | 5’- CCATAAAACATAACTAAAAACTAAA -3' |  |  |  |  |
|  | inner | 5’- TTTGTTATATTGTTTGTGGAAAA -3' | 429 | 54 |  |  |
|  |  | 5’- CCATAAAACATAACTAAAAACTAAA -3' |  |  |  |  |

**References**

1. Cheng D, Guo Y, Li Z, Liu Y, Gao X, Gao Y, et al. Porcine induced pluripotent stem cells require LIF and maintain their developmental potential in early stage of embryos. PloS one. 2012;7(12):e51778. doi: 10.1371/journal.pone.0051778. PubMed PMID: 23251622; PubMed Central PMCID: PMC3522612.

2. Zhao MT, Rivera RM, Prather RS. Locus-specific DNA methylation reprogramming during early porcine embryogenesis. Biology of reproduction. 2013;88(2):48. doi: 10.1095/biolreprod.112.104471. PubMed PMID: 23303676; PubMed Central PMCID: PMC3589235.

3. Hwang JY, Kim EB, Ka H, Lee CK. Identification of the porcine XIST gene and its differential CpG methylation status in male and female pig cells. PloS one. 2013;8(9):e73677. doi: 10.1371/journal.pone.0073677. PubMed PMID: 24040022; PubMed Central PMCID: PMC3767593.
